# Association of Family Meals and Prediabetes Among South Korean Adults Without Diabetes

**DOI:** 10.1155/jdr/7457214

**Published:** 2026-10-14

**Authors:** Eun-Ji Jung, Jae Hyeok Lim, Kui Son Choi, Min Jin Ha, Eun-Cheol Park

**Affiliations:** ^1^ Department of Healthcare Management Graduate School of Public Health Yonsei University Seoul Republic of Korea yonsei.ac.kr; ^2^ Department of Public Health Graduate School Yonsei University Seoul Republic of Korea yonsei.ac.kr; ^3^ Institute of Health Services Research Yonsei University Seoul Republic of Korea yonsei.ac.kr; ^4^ Department of Public Health and AI Graduate School of Cancer Science and Policy National Cancer Center Goyang Republic of Korea ncc.re.kr; ^5^ Department of Health Informatics and Biostatistics Graduate School of Public Health Yonsei University Seoul Republic of Korea yonsei.ac.kr; ^6^ Department of Preventive Medicine Yonsei University College of Medicine Seoul Republic of Korea yonsei.ac.kr

**Keywords:** blood glucose, family meals, prediabetes, Type 2 diabetes mellitus

## Abstract

**Introduction:**

Prediabetes is a reversible yet often overlooked condition that represents a critical window for early intervention. Family meal practices may support effective disease management during this stage. In this study, we aimed to investigate the association between family meal practices and the prevalence of prediabetes among South Korean adults.

**Methods:**

Data from the 2019–2023 Korea National Health and Nutrition Examination Survey were analyzed. Family meal practices were defined as sharing at least one meal (breakfast, lunch, or dinner) with family members three or more times per week. Prediabetes was defined as fasting glucose levels of 100–125 mg/dL or glycated hemoglobin of 5.7%–6.4%. Sex‐stratified multivariable logistic regression analyses were performed, adjusting for covariates.

**Results:**

Among 16,957 adults without diabetes (6907 men and 10,050 women), the prevalence of prediabetes was 49.1% in men and 42.3% in women. Men who participated in family meals had 16% lower odds of prediabetes (odds ratio [OR]: 0.84, 95% confidence interval [CI]: 0.73–0.97) compared with men who did not, whereas the association was not significant for women (OR: 0.97, 95% CI: 0.84–1.11). Among men, this association tended to be more pronounced in socioeconomic subgroups characterized by urban residence, higher household income, higher educational level, white‐collar occupations, and married status.

**Conclusions:**

Regular participation in family meals was associated with a lower prevalence of prediabetes among South Korean adult men without diabetes. This finding may help identify population subgroups that could benefit from targeted lifestyle‐based interventions for diabetes prevention.

## 1. Introduction

Diabetes mellitus, characterized by insufficient pancreatic insulin production or impaired insulin utilization, has substantially increased in global prevalence, rising from approximately 200 million individuals in 1990 to 830 million in 2022 [1]. South Korea also faces a substantial burden, with diabetes affecting an estimated 13.1% of adults aged ≥ 19 years (15.2% men, 11.0% women) and prediabetes (intermediate hyperglycemia) affecting 36.1% (38.3% men, 34.0% women) in 2021–2022 [2]. Prediabetes, an early stage in the development of Type 2 diabetes, is associated with an increased risk of progression to diabetes and diabetes‐related complications, yet many affected individuals remain unrecognized [3–5]. Early identification and intervention during this potentially modifiable stage may help prevent or delay the onset of diabetes and related complications [6].

Prediabetes shares many risk factors with Type 2 diabetes, and lifestyle interventions targeting weight management and dietary behaviors can reduce the risk of disease progression [7–9]. As these strategies require sustained behavioral changes and are embedded within family and social contexts, family‐based approaches have been increasingly proposed [10, 11]. Specifically, dietary behaviors are often shared among family members, as meals are typically consumed together within the household [12]. Unlike family‐based dietary interventions for diabetes management, which are guided by explicit therapeutic intentions, family meal practices among individuals with normoglycemia or prediabetes may instead reflect habitual social and dietary environments, particularly because prediabetes is often unrecognized or perceived as less serious [13].

Nevertheless, the potential benefits of family‐based dietary interventions suggest plausible pathways through which family meal practices may be already associated with glycemic health among individuals without diagnosed diabetes [14]. Shared family meals may promote healthier food choices and regular eating patterns while fostering family interaction and psychosocial support, which may in turn influence metabolic health and glucose regulation [15, 16]. Previous studies have primarily focused on the role of family meals in relation to the risk for Type 2 diabetes [17], diabetes management [18], or dietary behaviors among children and adolescents with diabetes, whose eating patterns are strongly shaped by parental practices [19, 20]. Thus, it remains unclear whether habitual family meal practices among adults without diabetes are associated with prediabetes in the absence of intentional disease‐management behaviors [21].

Given its high population prevalence and potential reversibility, prediabetes represents an important target for population‐level prevention [22]. However, family meal practices may vary according to how frequently and when meals are shared, as well as broader socioeconomic circumstances. Moreover, previous research suggests that associations between dietary patterns and metabolic outcomes may differ between men and women, potentially reflecting interactions between biological and sociocultural factors [23, 24]. Sex differences in dietary behaviors and domestic and food‐related responsibilities may contribute to such heterogeneity across diverse cultural contexts, including Korean households, where these responsibilities remain sex‐specific [25, 26]. Accordingly, we aimed to examine the association between family meal practices and the prevalence of prediabetes among adults without diabetes in South Korea and assessed whether these associations differed by sex.

## 2. Methods

### 2.1. Data

Data were obtained from the Korean National Health and Nutrition Examination Survey (KNHANES), a nationwide survey administered by the Korea Disease Control and Prevention Agency. The KNHANES uses a complex, multistage sampling design incorporating stratification and clustering to generate a nationally representative sample of the noninstitutionalized South Korean population. Participants are recruited from 254 geographically defined survey districts across South Korea, ensuring broad population coverage. Owing to its population‐based sampling framework, KNHANES data are widely considered representative of the general population of South Korea. As the KNHANES dataset is publicly accessible and all participant information is collected and released in anonymized form, institutional review board approval was not required for this study.

### 2.2. Participants

Using KNHANES data collected over five survey cycles (2019–2023), 23,100 adults were initially identified after excluding individuals with diabetes‐related conditions or missing information regarding diabetes status (*n* = 4284; Figure S1). The exclusion criteria were physician‐diagnosed diabetes, laboratory measurements that met the diagnostic criteria for diabetes, current use of glucose‐lowering medications or insulin therapy, and pregnancy. These exclusions were applied to minimize the influence of strong glycemic determinants unrelated to the exposures of interest. We further excluded 2819 participants with missing dietary intake or meal‐related information, resulting in an analytic sample of 20,281 individuals. An additional 3324 participants were excluded due to missing covariate data used in the analyses, and no imputation was performed. The final study population included 16,597 adults.

### 2.3. Variables

#### 2.3.1. Prediabetes

The dependent variable was prediabetes, defined according to fasting glucose (FG) and glycated hemoglobin (HbA1c) levels. Blood samples in KNHANES were collected after at least 8 h of fasting. FG was measured using the hexokinase method with a Labospect 008AS analyzer (Hitachi, Japan) in 2019–2021 and a Cobas 8000 analyzer (Roche, Germany) in 2022–2023. HbA1c was measured by high‐performance liquid chromatography using a Tosoh G8 analyzer (Tosoh, Japan) in 2019–2021 and a Tosoh HLC‐723G11 analyzer (Tosoh, Japan) in 2022–2023. Normoglycemia was defined as an FG level < 100 mg/dL and HbA1c level < 5.7%, whereas prediabetes was defined as an FG level of 100–125 mg/dL or HbA1c level of 5.7%–6.4%. These definitions were derived from the criteria of the American Diabetes Association [27].

#### 2.3.2. Family Meal Practices

Family meal practices, the variable of interest, were defined as sharing at least one of three daily meals (breakfast, lunch, and dinner) with family members. Each daily meal type was assessed separately, and participants were considered to have consumed a specific meal if they reported eating it at least three times per week during the previous year.

To further characterize family meal practices, we constructed additional variables capturing the frequency (none, once, twice, or three times) and timing (categorized as no family meals, breakfast only, lunch only, dinner only, breakfast and lunch, breakfast and dinner, lunch and dinner, and all meals) of family meals. These additional classifications were used to explore potentially distinct associations according to specific family meal practice patterns.

#### 2.3.3. Covariates

A conceptual framework illustrating the potential pathways linking family meal practices with prediabetes is presented in Figure S2. Candidate covariates were selected a priori as potential confounders that could influence the association between family meal practices and prediabetes. The final inclusion of covariates was guided by a stepwise variable selection procedure with prediabetes as the outcome. Demographic and socioeconomic variables included age, region, household income, educational level, marital status, and occupation. Health‐related behaviors included smoking status, drinking frequency, physical activity, and body mass index. Clinical factors potentially associated with prediabetes included hypertension, dyslipidemia, and a family history of diabetes (defined as having at least one first‐degree relative [father, mother, or sibling] with physician‐diagnosed diabetes). As multiple survey years were pooled, the survey year was included to account for temporal variation. Multicollinearity was assessed using variance inflation factors (VIF), with maximum VIFs of 1.93 among men and 1.98 among women, indicating no substantial multicollinearity.

### 2.4. Statistical Analyses

All analyses were stratified by sex because prior literature and the sociocultural context of dietary practices in South Korea suggest sex‐specific mechanisms underlying the association between family meal practices and prediabetes. Descriptive statistics are presented as frequencies and percentages according to prediabetes status. Differences between groups were assessed using chi‐square tests. To examine the association between family meal practices and prediabetes, multivariable logistic regression models were fitted with adjustment for prespecified demographic, socioeconomic, health‐related behavioral, clinical, and survey year covariates. Additional subgroup analyses were conducted by stratifying the sample according to selected covariates to explore potential effect modification. To further investigate which glycemic indicator primarily drove classification of prediabetes, the dependent variable was categorized into multiple mutually exclusive groups, and multinomial logistic regression models were used to account for its multinomial nature. The associations between detailed classifications of family meal practices and prediabetes were also examined. As sensitivity analyses, we compared nutrient intakes according with family meal practices using 24‐h dietary recall data to explore potential dietary differences underlying the observed associations. We also examined changes in the associations with sequential adjustment for different domains of covariates and conducted stratified analyses according to whether the 2020–2021 period, during which dietary patterns may have been affected by the COVID‐19 pandemic, was included in the study period. Adjusted odds ratios (ORs) and corresponding 95% confidence intervals (CIs) were estimated, and all analyses incorporated the complex survey design of the KNHANES, including survey‐specific strata, clusters, and sampling weights. Significance was defined as a two‐sided *p* value < 0.05. All analyses were performed using SAS software, Version 9.4 M7 (SAS Institute, Cary, North Carolina, United States).

## 3. Results

### 3.1. Descriptive Analyses

The general characteristics of the study participants, stratified by sex and prediabetes status, are presented in Table 1. In total, 16,957 adults without diabetes (6907 men and 10,050 women) were included in the study. Prediabetes was identified in 3388 men (49.1%) and 4249 women (42.3%). Regarding family meals, 4900 men (70.9%) and 7270 women (72.3%) reported having at least one meal per day with family members. In unadjusted comparisons, men who had family meals showed a higher proportion of prediabetes than those who did not, whereas among women, a higher proportion of prediabetes was observed in those who did not have family meals. These differences were examined using chi‐square tests and were significant for both men (*p* < 0.0001) and women (*p* = 0.0112). Substantial differences in prediabetes prevalence were observed among age groups, with higher age groups demonstrating progressively higher prevalence.

**Table 1:** General characteristics of the study population.

Variables	Prediabetes													
	Men							Women						
	Total		Yes		No		*p*	Total		Yes		No		*p*
	*N*	%	*N*	%	*N*	%		*N*	%	*N*	%	*N*	%	
Total (*N* = 16,957)	6907	100.0	3388	49.1	3519	50.9		10,050	100.0	4249	42.3	5801	57.7	
Family meals							< 0.0001							0.0112
No	2007	29.1	895	44.6	1112	55.4		2780	27.7	1232	44.3	1548	55.7	
Yes	4900	70.9	2493	50.9	2407	49.1		7270	72.3	3017	41.5	4253	58.5	
Age							< 0.0001							< 0.0001
−29	1030	14.9	162	15.7	868	84.3		1193	11.9	116	9.7	1077	90.3	
30–39	1028	14.9	375	36.5	653	63.5		1396	13.9	269	19.3	1127	80.7	
40–49	1237	17.9	597	48.3	640	51.7		1932	19.2	561	29.0	1371	71.0	
50–59	1133	16.4	672	59.3	461	40.7		2024	20.1	1060	52.4	964	47.6	
60–69	1261	18.3	785	62.3	476	37.7		1988	19.8	1223	61.5	765	38.5	
70+	1218	17.6	797	65.4	421	34.6		1517	15.1	1020	67.2	497	32.8	
Region							< 0.0001							< 0.0001
Metropolitan	3133	45.4	1455	46.4	1678	53.6		4582	45.6	1731	37.8	2851	62.2	
Urban	1531	22.2	746	48.7	785	51.3		2367	23.6	1028	43.4	1339	56.6	
Rural	2243	32.5	1187	52.9	1056	47.1		3101	30.9	1490	48.0	1611	52.0	
Household income							< 0.0001							< 0.0001
High	2270	32.9	1035	45.6	1235	54.4		2988	29.7	1096	36.7	1892	63.3	
Middle‐high	1977	28.6	941	47.6	1036	52.4		2800	27.9	1057	37.8	1743	62.3	
Middle‐low	1581	22.9	833	52.7	748	47.3		2479	24.7	1079	43.5	1400	56.5	
Low	1079	15.6	579	53.7	500	46.3		1783	17.7	1017	57.0	766	43.0	
Education level							< 0.0001							< 0.0001
College or above	3134	45.4	1410	45.0	1724	55.0		4066	40.5	1172	28.8	2894	71.2	
High school	2445	35.4	1142	46.7	1303	53.3		3290	32.7	1353	41.1	1937	58.9	
Middle school or below	1328	19.2	836	63.0	492	37.0		2694	26.8	1724	64.0	970	36.0	
Marital status							< 0.0001							< 0.0001
Married	4718	68.3	2656	56.3	2062	43.7		6659	66.3	2973	44.6	3686	55.4	
Unmarried, separated, widowed, divorced	2189	31.7	732	33.4	1457	66.6		3391	33.7	1276	37.6	2115	62.4	
Occupation							< 0.0001							< 0.0001
None	1966	28.5	950	48.3	1016	51.7		4625	46.0	2089	45.2	2536	54.8	
White‐collar	2079	30.1	915	44.0	1164	56.0		2455	24.4	678	27.6	1777	72.4	
Pink‐collar	694	10.0	309	44.5	385	55.5		1516	15.1	650	42.9	866	57.1	
Blue‐collar	2168	31.4	1214	56.0	954	44.0		1454	14.5	832	57.2	622	42.8	
Smoking status							< 0.0001							0.0001
None	2115	30.6	843	39.9	1272	60.1		9068	90.2	3892	42.9	5176	57.1	
Past	1945	28.2	980	50.4	965	49.6		433	4.3	170	39.3	263	60.7	
Current	2847	41.2	1565	55.0	1282	45.0		549	5.5	187	34.1	362	65.9	
Drinking frequency							< 0.0001							< 0.0001
No	1293	18.7	695	53.8	598	46.2		3542	35.2	1865	52.7	1677	47.3	
1–4 times in a month	3523	51.0	1538	43.7	1985	56.3		5382	53.6	1992	37.0	3390	63.0	
Several times in a week	2091	30.3	1155	55.2	936	44.8		1126	11.2	392	34.8	734	65.2	
Physical activity							< 0.0001							< 0.0001
No	3543	51.3	1915	54.1	1628	45.9		5756	57.3	2641	45.9	3115	54.1	
Yes	3364	48.7	1473	43.8	1891	56.2		4294	42.7	1608	37.4	2686	62.6	
Body mass index							< 0.0001							< 0.0001
Underweight	182	2.6	59	32.4	123	67.6		568	5.7	100	17.6	468	82.4	
Normal	3883	56.2	1704	43.9	2179	56.1		6693	66.6	2524	37.7	4169	62.3	
Obesity	2842	41.1	1625	57.2	1217	42.8		2789	27.8	1625	58.3	1164	41.7	
Hypertension							< 0.0001							< 0.0001
Normal	2918	42.2	1047	35.9	1871	64.1		5731	57.0	1694	29.6	4037	70.4	
Pre‐hypertension	1815	26.3	925	51.0	890	49.0		1706	17.0	867	50.8	839	49.2	
Hypertension	2174	31.5	1416	65.1	758	34.9		2613	26.0	1688	64.6	925	35.4	
Dyslipidemia							< 0.0001							< 0.0001
No	4554	65.9	1927	42.3	2627	57.7		6758	67.2	2197	32.5	4561	67.5	
Yes	2353	34.1	1461	62.1	892	37.9		3292	32.8	2052	62.3	1240	37.7	
Family history of diabetes							0.0087							< 0.0001
No	5521	79.9	2664	48.3	2857	51.7		7556	75.2	3064	40.6	4492	59.4	
Yes	1386	20.1	724	52.2	662	47.8		2494	24.8	1185	47.5	1309	52.5	
Year							< 0.0001							< 0.0001
2019	1502	21.7	813	54.1	689	45.9		2214	22.0	1043	47.1	1171	52.9	
2020	1177	17.0	629	53.4	548	46.6		1682	16.7	770	45.8	912	54.2	
2021	1288	18.6	711	55.2	577	44.8		1873	18.6	981	52.4	892	47.6	
2022	1415	20.5	623	44.0	792	56.0		2014	20.0	708	35.2	1306	64.8	
2023	1525	22.1	612	40.1	913	59.9		2267	22.6	747	33.0	1520	67.0	

### 3.2. Regression Analyses

The associations between family meal practices and prediabetes are presented in Table 2. After multivariable adjustment, men who had meals with family members had a significantly lower likelihood of prediabetes than those who did not (OR: 0.84, 95% CI: 0.73–0.97), corresponding to a 16% reduction in odds. Among women, family meal participation was associated with 3% lower odds of prediabetes; however, this association was not significant (OR: 0.97, 95% CI: 0.84–1.11).

**Table 2:** Results of factors associated between family meals and prediabetes.

Variables	Prediabetes			
	Men		Women	
	OR	95% CI	OR	95% CI
Family meals				
No	1.00		1.00	
Yes	0.84	(0.73–0.97)	0.97	(0.84–1.11)
Age				
−29	1.00		1.00	
30–39	2.61	(2.00–3.39)	1.93	(1.47–2.55)
40–49	3.59	(2.71–4.75)	2.74	(2.12–3.54)
50–59	5.58	(4.21–7.40)	6.12	(4.69–8.00)
60–69	7.50	(5.58–10.08)	8.89	(6.77–11.68)
70+	10.79	(7.76–15.01)	11.26	(8.26–15.37)
Region				
Metropolitan	1.00		1.00	
Urban	1.01	(0.86–1.19)	1.09	(0.95–1.25)
Rural	1.07	(0.91–1.26)	1.16	(1.01–1.33)
Household income				
High	1.00		1.00	
Middle‐high	0.99	(0.85–1.16)	0.95	(0.83–1.10)
Middle‐low	1.04	(0.88–1.23)	0.89	(0.76–1.04)
Low	0.78	(0.62–0.99)	0.99	(0.81–1.22)
Education level				
College or above	1.00		1.00	
High school	1.10	(0.94–1.28)	1.06	(0.92–1.21)
Middle school or below	0.99	(0.79–1.24)	1.02	(0.84–1.24)
Marital status				
Married	1.00		1.00	
Unmarried, separated, widowed, divorced	0.88	(0.73–1.05)	0.91	(0.79–1.04)
Occupation				
None	1.00		1.00	
White‐collar	1.04	(0.85–1.27)	1.12	(0.96–1.31)
Pink‐collar	1.05	(0.82–1.34)	1.14	(0.97–1.34)
Blue‐collar	1.20	(1.01–1.44)	1.28	(1.09–1.50)
Smoking status				
None	1.00		1.00	
Past	1.19	(1.00–1.41)	1.21	(0.90–1.61)
Current	1.08	(0.92–1.26)	0.97	(0.76–1.23)
Drinking frequency				
No	1.00		1.00	
1–4 times in a month	1.04	(0.88–1.24)	0.95	(0.84–1.08)
Several times in a week	1.17	(0.97–1.40)	0.86	(0.71–1.05)
Physical activity				
No	1.00		1.00	
Yes	0.87	(0.77–0.98)	0.90	(0.81–1.01)
Body mass index				
Underweight	1.00		1.00	
Normal	1.70	(1.12–2.57)	1.58	(1.20–2.07)
Obesity	3.34	(2.20–5.07)	3.41	(2.56–4.54)
Hypertension				
Normal	1.00		1.00	
Pre‐hypertension	1.31	(1.13–1.51)	1.33	(1.14–1.55)
Hypertension	1.51	(1.30–1.75)	1.51	(1.31–1.74)
Dyslipidemia				
No	1.00		1.00	
Yes	1.57	(1.38–1.79)	1.91	(1.69–2.16)
Family history of diabetes				
No	1.00		1.00	
Yes	1.20	(1.04–1.39)	1.63	(1.43–1.85)
Year				
2019	1.00		1.00	
2020	0.95	(0.76–1.18)	1.01	(0.84–1.21)
2021	0.97	(0.79–1.20)	1.26	(1.06–1.49)
2022	0.60	(0.50–0.73)	0.48	(0.40–0.58)
2023	0.49	(0.41–0.59)	0.42	(0.36–0.50)

*Note:* Adjusted for demographic, socioeconomic, health‐related behavioral, clinical, and survey year covariates.

The associations varied according to covariate adjustment (Table S1). In the unadjusted model, the OR was 1.24 (95% CI, 1.10–1.40) among men and 0.97 (95% CI, 0.87–1.08) among women. Among men, adjustment for demographic and socioeconomic covariates changed the direction of the association, with an OR of 0.83 (95% CI, 0.72–0.96). The OR remained below 1.00 after further adjustment for health‐related behaviors covariates (Model 3) and all prespecified covariates (Model 4).

### 3.3. Subgroup and Additional Analyses

The results of subgroup analyses stratified by independent variables (covariates) are presented in Table 3. In contrast to women, several subgroups of men demonstrated a more pronounced association between family meal participation and prediabetes, predominantly across indicators of socioeconomic status. Specifically, family meal practices were associated with a significantly lower likelihood of prediabetes among men residing in urban areas (OR: 0.59, 95% CI: 0.43–0.81); those with higher household income (OR: 0.71, 95% CI: 0.55–0.91), higher educational levels (OR: 0.76, 95% CI: 0.62–0.94), and married status (OR: 0.75, 95% CI: 0.62–0.91); and those employed in white‐collar occupations (OR: 0.70, 95% CI: 0.54–0.90). Additionally, a stronger effect size was observed among men with current dyslipidemia (OR: 0.73, 95% CI: 0.56–0.94).

**Table 3:** Results of subgroup analysis stratified by independent variables.

Variables	Prediabetes					
	Men			Women		
	No	Yes		No	Yes	
	OR	OR	95% CI	OR	OR	95% CI
Region						
Metropolitan	1.00	0.84	(0.68–1.03)	1.00	0.97	(0.79–1.19)
Urban	1.00	0.59	(0.43–0.81)	1.00	0.81	(0.61–1.08)
Rural	1.00	1.14	(0.88–1.49)	1.00	1.11	(0.85–1.44)
Household income						
High	1.00	0.71	(0.55–0.91)	1.00	0.97	(0.74–1.29)
Middle‐high	1.00	0.90	(0.69–1.17)	1.00	1.03	(0.79–1.34)
Middle‐low	1.00	0.91	(0.66–1.27)	1.00	0.82	(0.62–1.09)
Low	1.00	1.22	(0.81–1.84)	1.00	1.02	(0.74–1.40)
Education level						
College or above	1.00	0.76	(0.62–0.94)	1.00	1.22	(0.96–1.53)
High school	1.00	1.04	(0.81–1.33)	1.00	0.82	(0.64–1.06)
Middle school or below	1.00	0.83	(0.55–1.24)	1.00	0.93	(0.72–1.21)
Marital status						
Married	1.00	0.75	(0.62–0.91)	1.00	0.90	(0.74–1.10)
Unmarried, separated, widowed, divorced	1.00	0.96	(0.76–1.23)	1.00	1.17	(0.94–1.45)
Occupation						
None	1.00	1.20	(0.87–1.66)	1.00	1.04	(0.84–1.28)
White‐collar	1.00	0.70	(0.54–0.90)	1.00	1.11	(0.82–1.51)
Pink‐collar	1.00	0.70	(0.44–1.09)	1.00	0.80	(0.57–1.12)
Blue‐collar	1.00	0.93	(0.70–1.22)	1.00	0.70	(0.48–1.03)
Dyslipidemia						
No	1.00	0.92	(0.76–1.10)	1.00	0.91	(0.75–1.09)
Yes	1.00	0.73	(0.56–0.94)	1.00	1.08	(0.86–1.35)
Family history of diabetes						
No	1.00	0.84	(0.72–0.99)	1.00	1.00	(0.84–1.18)
Yes	1.00	0.82	(0.61–1.10)	1.00	0.88	(0.67–1.15)
Year						
2019	1.00	0.73	(0.52–1.03)	1.00	0.93	(0.69–1.27)
2020	1.00	0.86	(0.61–1.21)	1.00	1.13	(0.79–1.61)
2021	1.00	1.00	(0.70–1.42)	1.00	1.04	(0.75–1.44)
2022	1.00	0.95	(0.70–1.27)	1.00	1.08	(0.79–1.47)
2023	1.00	0.78	(0.58–1.06)	1.00	0.70	(0.52–0.93)

*Note:* Adjusted all covariates except for each stratified variable.

The results of multinomial logistic regression analyses, stratified by the different indicators used to define prediabetes, are presented in Table 4. Among men, family meal participation was associated with lower odds of prediabetes defined by FG, HbA1c, and the combined criteria. Among them, the association was the strongest and most significant for FG‐defined prediabetes (OR: 0.80, 95% CI: 0.65–0.98).

**Table 4:** Results of analysis stratified by prediabetes.

Variables		Prediabetes						
		No	Fasting glucose		HbA1c		Both	
		OR	OR	95% CI	OR	95% CI	OR	95% CI
Men	Family meals							
	No	1.00						
	Yes		0.80	(0.65–0.98)	0.89	(0.72–1.11)	0.85	(0.69–1.04)
Women	Family meals							
	No	1.00						
	Yes		1.10	(0.89–1.37)	0.92	(0.76–1.11)	0.91	(0.75–1.11)

*Note:* Results of the multinomial logistic regression adjusted for demographic, socioeconomic, health‐related behavioral, clinical, and survey year covariates.

Additional analyses examining the frequency and timing of family meals are displayed in Table 5. Among men, the most pronounced association was observed in those who had family meals twice a day (OR: 0.75, 95% CI: 0.63–0.90); however, no statistically significant trend was observed with increasing family meal frequency (*p* for trend = 0.0938). Regarding meal timing, significant associations were observed for family meals consumed at breakfast (OR: 0.67, 95% CI: 0.48–0.94), at both breakfast and dinner (OR: 0.77, 95% CI: 0.63–0.95), and at both lunch and dinner (OR: 0.68, 95% CI: 0.51–0.92).

**Table 5:** Results of analysis stratified by frequency and timing of family meals.

Variables	Prediabetes					
	Men			Women		
	*N*	OR	95% CI	*N*	OR	95% CI
Frequency of family meals						
None	2007	1.00		2780	1.00	
1 time	2290	0.86	(0.74–1.01)	3377	0.99	(0.84–1.15)
2 times	1499	0.75	(0.63–0.90)	2,440	0.97	(0.83–1.14)
3 times	1111	0.97	(0.77–1.22)	1453	0.89	(0.73–1.08)
Family meal timing						
No family meals	2007	1.00		2780	1.00	
Breakfast	262	0.67	(0.48–0.94)	335	0.87	(0.64–1.17)
Lunch	83	0.91	(0.53–1.54)	149	1.44	(0.93–2.22)
Dinner	1945	0.89	(0.76–1.05)	2893	0.98	(0.83–1.15)
Breakfast and lunch	50	0.85	(0.44–1.65)	88	1.29	(0.72–2.31)
Breakfast and dinner	1083	0.77	(0.63–0.95)	1722	1.01	(0.84–1.20)
Lunch and dinner	366	0.68	(0.51–0.92)	630	0.85	(0.66–1.09)
Every meal	1111	0.97	(0.77–1.22)	1453	0.89	(0.73–1.08)

*Note: p*‐for trend across increasing frequency of family meals was 0.0938 among men and 0.2964 among women. Adjusted for demographic, socioeconomic, health‐related behavioral, clinical, and survey year covariates.

Among men, those who had family meals consumed higher amounts of carbohydrates, dietary fiber, potassium, and magnesium and a lower amount of total fat than those who did not have family meals. The lower total fat intake was not observed among women (Table S2). In analyses stratified by study period, the association among men was no longer statistically significant during 2020–2021, whereas a stronger inverse association was observed during 2019 and 2022–2023 (OR, 0.80; 95% CI, 0.66–0.95) (Table S3).

## 4. Discussion

In this study, we investigated the association between family meal practices and the prevalence of prediabetes among South Korean adults. Our findings indicate that among South Korean adults without diabetes, men who shared at least one meal per day with family members had a lower likelihood of prediabetes than those who did not; the same was not observed for women. The pronounced associations observed across subgroups stratified by residential area, income level, educational level, marital status, and employment status suggest that a complex interplay of socioeconomic factors influences this relationship. Furthermore, the association was more pronounced when prediabetes was defined by FG levels and among those sharing two daily family meals, clarifying the glycemic context and subgroup in which this relationship was most evident.

Previous research on family‐based dietary practices in diabetes has largely focused on individuals diagnosed with diabetes, and on children or adolescents (whose eating behaviors are strongly shaped by parental habits), and has generally reported associations with healthier dietary patterns and improved diabetes‐related outcomes [18–20, 28–30]. However, relatively few studies have examined the association between family meal practices and prediabetes, a stage characterized by a high risk for progression to diabetes [5]. In this context, our findings extend the existing literature and indicate that the association between family meal practices and prediabetes prevalence may differ by sex after covariate adjustment.

Among men, demographic and socioeconomic characteristics, particularly age and marital status, appeared to account for much of the crude association between family meal practices and prediabetes, with the association attenuating and changing direction after adjustment. This pattern suggests that family meal practices may partly serve as a proxy for underlying demographic and socioeconomic characteristics, and highlights a qualitatively different pattern of confounding by sex [31, 32]. This sex‐specific pattern aligns with prior studies reporting associations between family meals and lower triglyceride and FG levels among men only [33]. One possible explanation is that the potential benefits of family meal practices may operate partly through healthier dietary behaviors and related lifestyle factors, including those associated with adiposity, and these factors may differ by sex [23, 34]. In the South Korean context, women have traditionally assumed a greater share of domestic responsibilities, particularly meal planning and preparation, which may contribute to the dietary practices of married, working‐age men who participate in family meals [35, 36]. Conversely, women may be more likely to maintain dietary patterns characterized by higher fiber and vegetable intake and more structured meal routines regardless of family meal participation, potentially reducing the additional association attributable to family meal practices [37].

The subgroup analyses stratified by socioeconomic factors demonstrated that urban residents who have a higher household income and educational attainment, are married, and work in office‐based occupations may be more influenced by family meal practices. These patterns may reflect pathways through which dietary behaviors contribute to socioeconomic disparities in the burden of chronic conditions, such as diabetes [38, 39]. Individuals with higher socioeconomic status have greater health awareness and better access to high‐quality foods, increasing the likelihood that nutritionally balanced and higher‐quality dietary components are incorporated into daily meals [40]. Particularly, an appropriate level of dietary fat intake may have more favorable insulin sensitivity among men [41]. In this context, the observed associations may not merely reflect the act of sharing meals, but rather the provision of relatively well‐managed diets during meals shared with a spouse, specifically among men.

Additionally, in subgroups with conditions requiring lifestyle modification (such as dyslipidemia), intentional participation in family meals may serve as a dietary management strategy, which may partly explain the significant associations observed in these populations. The clinical interpretation of FG and HbA1c as indicators of prediabetes differs depending on the underlying glycemic dynamics [27]. The stronger association observed for FG may suggest that, among men, potential benefits of the regular dietary and daily routines associated with family meal practices may be more readily reflected in FG than in HbA1c, which represents longer‐term glycemic exposure [42]. As FG may be more sensitive to short‐term fluctuations in glycemic regulation, these potential benefits may be apparent during the early stage of prediabetes, when glucose regulation remains relatively dynamic [7]. Further investigation is warranted to clarify the potential differences in the responses of these two biomarkers to family meal practices. In South Korea, working men are more likely to share breakfast or dinner with family members; the larger effect sizes observed for two daily family meals, particularly those including breakfast and dinner, may be interpreted considering this social pattern [43].

By leveraging nationally representative data, this study examined the association between family meal practices and biomarker‐defined prediabetes. These findings provide population‐level evidence relevant to lifestyle‐based approaches to diabetes prevention. Nonetheless, certain limitations of this study should be acknowledged. First, the cross‐sectional design precludes any causal inference regarding the relationship between family meal participation and prediabetes. Second, information on family meals and several lifestyle‐related covariates was collected via self‐reported questionnaires, which may be subject to recall bias. Third, despite adjustment for multiple confounders, residual confounding by unmeasured or inadequately measured factors related to both family meal practices and prediabetes cannot be ruled out. Fourth, the study period encompassed the coronavirus disease pandemic (2020–2021), during which substantial social changes, such as the expansion of remote work, reduced dining out, and increased home‐based meals, may have temporarily influenced family meal patterns. To assess the potential influence of the pandemic, we conducted sex‐stratified sensitivity analyses separately for 2020–2021 and for 2019 and 2022–2023; nevertheless, the broader contextual changes during the pandemic should be considered when interpreting the findings. Finally, the study population was limited to South Korean adults aged ≥ 19 years; therefore, the generalizability of the results to adolescents, populations in other countries, or individuals from different sociocultural backgrounds, including multicultural households, is limited. Future studies incorporating diverse age groups and cultural contexts, as well as longitudinal designs, are warranted to clarify the causal relationship between family meals and metabolic disorders.

## 5. Conclusions

Our findings suggest that among South Korean adult men without diabetes, those who regularly shared meals with their family had a lower likelihood of prediabetes, particularly when defined by FG levels, compared with those who did not. Additionally, the characteristics of this at‐risk group appear to be associated with socioeconomic factors such as urban residence, higher income, higher educational level, white‐collar occupations, and being married. These findings may help identify population subgroups that could benefit from targeted lifestyle‐based strategies to prevent chronic metabolic diseases.

## Author Contributions

E.J‐J. was responsible for conceptualization, investigation, project administration, writing the original draft, and reviewing and editing the manuscript. J.H.L. was responsible for data curation, formal analysis, investigation, visualization, and manuscript review and editing. K.S.C. was responsible for methodology and supervision. M.J.H. was responsible for methodology and validation. E‐C.P. was responsible for conceptualization, formal analysis, methodology, resources, and writing the original draft. E.J‐J. and J.H.L. have contributed to the work equally and should be regarded as co‐first authors.

## Funding

No funding was received for this manuscript.

## Disclosure

All authors have read and approved the final manuscript. J.H.L. had full access to all of the data in this study and takes complete responsibility for the integrity of the data and the accuracy of the data analysis.

## Ethics Statement

The Korean National Health and Nutrition Examination Survey dataset is publicly accessible, and all participant information is collected and released in anonymized form; therefore, institutional review board approval and informed consent were not required for this study.

## Conflicts of Interest

The authors declare no conflicts of interest.

## Supporting information


**Supporting Information** Additional supporting information can be found online in the Supporting Information section. Figure S1: Flowchart of the study participants displaying the inclusion and exclusion. Figure S2: Conceptual framework illustrating potential pathways and covariates in the association between family meal practices and prediabetes. Table S1: Results of factors associated with family meals and prediabetes. Table S2: Comparison of nutrient intake by family meal participation status. Table S3: Sensitivity analysis stratified by inclusion of the COVID‐19 period (2020 and 2021).

## Data Availability

The data that support the findings of this study are available from the corresponding author upon reasonable request.

## References

[1] World Health Organization , Diabetes, 2026, https://www.who.int/news-room/fact-sheets/detail/diabetes.

[2] Park S. E. , Ko S. H. , Kim J. Y. , Kim K. , Moon J. H. , Kim N. H. , Han K. D. , Choi S. H. , and Cha B. S. , Diabetes Fact Sheets in Korea 2024, Diabetes and Metabolism Journal. (2025) 49, no. 1, 24–33, 10.4093/dmj.2024.0818, 39828976. PMC11788544 39828976

[3] Bansal N. , Prediabetes Diagnosis and Treatment: A Review, World Journal of Diabetes. (2015) 6, no. 2, 296–303, 10.4239/wjd.v6.i2.296, 25789110. PMC4360422 25789110

[4] Tabák A. G. , Herder C. , Rathmann W. , Brunner E. J. , and Kivimäki M. , Prediabetes: A High-Risk State for Diabetes Development, Lancet. (2012) 379, no. 9833, 2279–2290, 10.1016/s0140-6736(12)60283-9, 22683128. PMC3891203 22683128

[5] Prakoso D. A. , Mahendradhata Y. , and Istiono W. , Family Involvement to Stop the Conversion of Prediabetes to Diabetes, Korean Journal of Family Medicine. (2023) 44, no. 6, 303–310, 10.4082/kjfm.23.0019, 37582666. PMC10667073 37582666

[6] Handelsman Y. , Bloomgarden Z. T. , Grunberger G. , Umpierrez G. , Zimmerman R. S. , Bailey T. S. , Blonde L. , Bray G. A. , Cohen A. J. , Dagogo-Jack S. , Davidson J. A. , Einhorn D. , Ganda O. P. , Garber A. J. , Garvey W. T. , Henry R. R. , Hirsch I. B. , Horton E. S. , Hurley D. L. , Jellinger P. S. , Jovanovič L. , Lebovitz H. E. , LeRoith D. , Levy P. , McGill J. B. , Mechanick J. I. , Mestman J. H. , Moghissi E. S. , Orzeck E. A. , Pessah-Pollack R. , Rosenblit P. D. , Vinik A. I. , Wyne K. , and Zangeneh F. , American Association of Clinical Endocrinologists and American College of Endocrinology--Clinical Practice Guidelines for Developing a Diabetes Mellitus Comprehensive Care Plan--2015--Executive Summary, Endocrine Practice. (2015) 21, no. 4, 413–437, 10.4158/EP15672.GL, 25869408. 27408942

[7] Unnikrishnan R. , Shaw J. E. , Chan J. C. N. , Wild S. H. , Peters A. L. , Orrange S. , Roden M. , and Mohan V. , Prediabetes, Nature Reviews Disease Primers. (2025) 11, no. 1, 10.1038/s41572-025-00635-0. 40676018

[8] Saito T. , Watanabe M. , Nishida J. , Izumi T. , Omura M. , Takagi T. , Fukunaga R. , Bandai Y. , Tajima N. , Nakamura Y. , Ito M. , and Zensharen Study for Prevention of Lifestyle Diseases Group , Lifestyle Modification and Prevention of Type 2 Diabetes in Overweight Japanese With Impaired Fasting Glucose Levels: A Randomized Controlled Trial, Archives of Internal Medicine. (2011) 171, no. 15, 1352–1360, 10.1001/archinternmed.2011.275, 21824948. 21824948

[9] García-Molina L. , Lewis-Mikhael A. M. , Riquelme-Gallego B. , Cano-Ibáñez N. , Oliveras-López M. J. , and Bueno-Cavanillas A. , Improving Type 2 Diabetes Mellitus Glycaemic Control Through Lifestyle Modification Implementing Diet Intervention: A Systematic Review and Meta-Analysis, European Journal of Nutrition. (2020) 59, no. 4, 1313–1328, 10.1007/s00394-019-02147-6, 31781857. 31781857

[10] Gillman M. W. , Rifas-Shiman S. L. , Frazier A. L. , Rockett H. R. , Camargo CA Jr , Field A. E. , Berkey C. S. , and Colditz G. A. , Family Dinner and Diet Quality Among Older Children and Adolescents, Archives of Family Medicine. (2000) 9, no. 3, 235–240, 10.1001/archfami.9.3.235, 10728109. 10728109

[11] Denham S. A. , Ware L. J. , Raffle H. , and Leach K. , Family Inclusion in Diabetes Education: A Nationwide Survey of Diabetes Educators, Diabetes Educator. (2011) 37, no. 4, 528–535, 10.1177/0145721711411312, 21690434. 21690434

[12] Robson S. M. , McCullough M. B. , Rex S. , Munafò M. R. , and Taylor G. , Family Meal Frequency, Diet, and Family Functioning: A Systematic Review With Meta-Analyses, Journal of Nutrition Education and Behavior. (2020) 52, no. 5, 553–564, 10.1016/j.jneb.2019.12.012, 31982371. 31982371

[13] O’Brien M. J. , Moran M. R. , Tang J. W. , Vargas M. C. , Talen M. , Zimmermann L. J. , Ackermann R. T. , and Kandula N. R. , Patient Perceptions About Prediabetes and Preferences for Diabetes Prevention, Diabetes Educator. (2016) 42, no. 6, 667–677, 10.1177/0145721716666678, 27621093. PMC5228412 27621093

[14] Evert A. B. , Dennison M. , Gardner C. D. , Garvey W. T. , Lau K. H. K. , MacLeod J. , Mitri J. , Pereira R. F. , Rawlings K. , Robinson S. , Saslow L. , Uelmen S. , Urbanski P. B. , and Yancy W. S. , Nutrition Therapy for Adults With Diabetes or Prediabetes: A Consensus Report, Diabetes Care. (2019) 42, no. 5, 731–754, 10.2337/dci19-0014, 31000505. PMC7011201 31000505

[15] Fruh S. M. , Fulkerson J. A. , Mulekar M. S. , Kendrick L. A. J. , and Clanton C. , The Surprising Benefits of the Family Meal, Journal for Nurse Practitioners. (2011) 7, no. 1, 18–22, 10.1016/j.nurpra.2010.04.017.

[16] Fernandez M. A. , Desroches S. , Marquis M. , and Provencher V. , Meal Planning as a Strategy to Support Healthy Eating, Nutrition Science en Évolution. (2020) 17, no. 3, 12–16, 10.7202/1068830ar.

[17] Zong G. , Eisenberg D. M. , Hu F. B. , and Sun Q. , Consumption of Meals Prepared at Home and Risk of Type 2 Diabetes: An Analysis of Two Prospective Cohort Studies, PLoS Medicine. (2016) 13, no. 7, e1002052, 10.1371/journal.pmed.1002052, 27379673. PMC4933392 27379673

[18] Baig A. A. , Benitez A. , Quinn M. T. , and Burnet D. L. , Family Interventions to Improve Diabetes Outcomes for Adults, Annals of the New York Academy of Sciences. (2015) 1353, no. 1, 89–112, 10.1111/nyas.12844, 26250784. PMC4624026 26250784

[19] Mahmood L. , González-Gil E. M. , Schwarz P. , Herrmann S. , Karaglani E. , Cardon G. , de Vylder F. , Willems R. , Makrilakis K. , Liatis S. , Iotova V. , Tsochev K. , Tankova T. , Rurik I. , Radó S. , Moreno L. A. , Manios Y. , on behalf of the Feel4Diabetes-Study Group , Manios Y. , Cardon G. , Lindström J. , Schwarz P. , Makrilakis K. , Annemans L. , Ko W. , Karatzi K. , Androutsos O. , Moschonis G. , Kanellakis S. , Mavrogianni C. , Tsoutsoulopoulou K. , Katsarou C. , Karaglani E. , Qira I. , Skoufas E. , Maragkopoulou K. , Tsiafitsa A. , Sotiropoulou I. , Tsolakos M. , Argyri E. , Nikolaou M. , Vampouli E. A. , Filippou C. , Apergi K. , Filippou A. , Katerina G. , Dimitriadis E. , Laatikainen T. , Wikström K. , Kivelä J. , Valve P. , Levälahti E. , Virtanen E. , Pennanen T. , Olli S. , Nelimarkka K. , van Stappen V. , Huys N. , Willems R. , Shadid S. , Timpel P. , Liatis S. , Dafoulas G. , Lambrinou C. P. , Giannopoulou A. , Karuranga E. , Moreno L. , Civeira F. , Bueno G. , de Miguel-Etayo P. , Gonzalez-Gil E. M. , Miguel-Berges M. L. , Giménez-Legarre N. , Flores-Barrantes P. , Ayala-Marín A. M. , Seral-Cortés M. , Baila-Rueda L. , Cenarro A. , Jarauta E. , Mateo-Gallego R. , Iotova V. , Tankova T. , Usheva N. , Tsochev K. , Chakarova N. , Galcheva S. , Dimova R. , Bocheva Y. , Radkova Z. , Marinova V. , Bazdarska Y. , Stefanova T. , Rurik I. , Ungvari T. , Jancsó Z. , Nánási A. , Kolozsvári L. , Semánova C. , Bíró É. , Antal E. , Radó S. , Martinez R. , and Tong M. , Frequency of Family Meals and Food Consumption in Families at High Risk of Type 2 Diabetes: The Feel4Diabetes-Study, European Journal of Pediatrics. (2022) 181, no. 6, 2523–2534, 10.1007/s00431-022-04445-4, 35353229. PMC9110493 35353229

[20] Kornides M. L. , Nansel T. R. , Quick V. , Haynie D. L. , Lipsky L. M. , Laffel L. M. B. , and Mehta S. N. , Associations of Family Meal Frequency With Family Meal Habits and Meal Preparation Characteristics Among Families of Youth With Type 1 Diabetes, Child: Care, Health and Development. (2014) 40, no. 3, 405–411, 10.1111/cch.12078, 23731337. PMC4086450 23731337

[21] Noronha J. C. , Nishi S. K. , Khan T. A. , Blanco Mejia S. , Kendall C. W. C. , Kahleová H. , Rahelić D. , Salas-Salvadó J. , Leiter L. A. , Lean M. E. J. , and Sievenpiper J. L. , Weight Management Using Meal Replacements and Cardiometabolic Risk Reduction in Individuals With Pre-Diabetes and Features of Metabolic Syndrome: A Systematic Review and Meta-Analysis of Randomized Controlled Trials, Obesity Reviews. (2024) 25, no. 7, e13751, 10.1111/obr.13751, 38693302. 38693302

[22] American Diabetes Association , 5. Prevention or Delay of Type 2 Diabetes:Standards of Medical Care in Diabetes-2018, Diabetes Care. (2018) 41, no. supplement 1, S51–S54, 10.2337/dc18-S005. 29222376

[23] Hur H. J. , Yang H. J. , Kim M. J. , Jang H. J. , Lee B. K. , Kim M. S. , and Park S. , Gender-Specific Associations of Diet Quality and Dietary Patterns With Metabolic Syndrome: Evidence From Korea National Health and Nutrition Examination Survey (KNHANES) VI, Nutrition & Metabolism. (2026) 23, no. 1, 10.1186/s12986-026-01116-1, 42032652. PMC13262307 42032652

[24] Chang S. H. , Chang Y. Y. , and Wu L. Y. , Gender Differences in Lifestyle and Risk Factors of Metabolic Syndrome: Do Women Have Better Health Habits Than Men?, Journal of Clinical Nursing. (2019) 28, no. 11-12, 2225–2234, 10.1111/jocn.14824. 30786102

[25] Noh J. W. , Kim K. B. , Park J. , Hong J. , and Kwon Y. D. , Relationship Between the Number of Family Members and Stress by Gender: Cross-Sectional Analysis of the Fifth Korea National Health and Nutrition Examination Survey, PLoS One. (2017) 12, no. 9, e0184235, 10.1371/journal.pone.0184235, 28886136. PMC5590891 28886136

[26] Rivera V. and Giacoman C. , Family Meals in Santiago de Chile: An Analysis of the Role of Family, Gender and Social Class in Commensality, Appetite. (2019) 140, 197–205, 10.1016/j.appet.2019.05.017, 31112738. 31112738

[27] American Diabetes Association , Diagnosis and classification of diabetes mellitus, Diabetes Care. (2014) 37, S81–S90, 10.2337/dc14-S081. 24357215

[28] Knowler W. C. , Barrett-Connor E. , Fowler S. E. , Hamman R. F. , Lachin J. M. , Walker E. A. , Nathan D. M. , and Diabetes Prevention Program Research Group , Reduction in the Incidence of Type 2 Diabetes With Lifestyle Intervention or Metformin, New England Journal of Medicine. (2002) 346, no. 6, 393–403, 10.1056/NEJMoa012512, 11832527. PMC1370926 11832527

[29] Vargas-Ortiz K. , Lira-Mendiola G. , Gómez-Navarro C. M. , Padilla-Estrada K. , Angulo-Romero F. , Hernández-Márquez J. M. , Villa-Martínez A. K. , González-Mena J. N. , Macías-Cervantes M. H. , Reyes-Escogido M. L. , and Guardado-Mendoza R. , Effect of a Family and Interdisciplinary Intervention to Prevent T2D: Randomized Clinical Trial, BMC Public Health. (2020) 20, no. 1, 10.1186/s12889-020-8203-1, 31969136. PMC6977289 31969136

[30] Choi S. E. , Diet-Specific Family Support and Glucose Control Among Korean Immigrants With Type 2 Diabetes, Diabetes Educator. (2009) 35, no. 6, 978–985, 10.1177/0145721709349220, 19934457. PMC3793646 19934457

[31] Tu Y. K. , Gunnell D. , and Gilthorpe M. S. , Simpson′s Paradox, Lord′s Paradox, and Suppression Effects Are the Same Phenomenon--The Reversal Paradox, Emerging Themes in Epidemiology. (2008) 5, no. 1, 10.1186/1742-7622-5-2, 18211676. PMC2254615 18211676

[32] Vetter T. R. and Mascha E. J. , Bias, Confounding, and Interaction: Lions and Tigers, and Bears, Oh My!, Anesthesia and Analgesia. (2017) 125, no. 3, 1042–1048, 10.1213/ane.0000000000002332, 28817531. 28817531

[33] Jeong T. W. , Jung M. J. , Lee J. W. , and Kim T. Y. , Effects of Family Meal Frequency on Risk Factors for Cardiovascular Disease in Korean Elderly Males and Females, Iranian Journal of Public Health. (2015) 44, no. 1, 60–67, 26060777. PMC4450015 26060777

[34] Lin L.-Y. , Hsu C.-Y. , Lee H.-A. , Tinkov A. A. , Skalny A. V. , Wang W. H. , and Chao J. C. J. , Gender Difference in the Association of Dietary Patterns and Metabolic Parameters With Obesity in Young and Middle-Aged Adults With Dyslipidemia and Abnormal Fasting Plasma Glucose in Taiwan, Nutrition Journal. (2019) 18, no. 1, 10.1186/s12937-019-0503-x, 31733645. PMC6858632 31733645

[35] Kim G. S. , Cho W. J. , Lee C. Y. , Marion L. N. , and Kim M. J. , The Relationship of Work Stress and Family Stress to the Self-Rated Health of Women Employed in the Industrial Sector in Korea, Public Health Nursing. (2005) 22, no. 5, 389–397, 10.1111/j.0737-1209.2005.220503.x, 16229731. 16229731

[36] Flagg L. A. , Sen B. , Kilgore M. , and Locher J. L. , The Influence of Gender, Age, Education and Household Size on Meal Preparation and Food Shopping Responsibilities, Public Health Nutrition. (2014) 17, no. 9, 2061–2070, 10.1017/s1368980013002267, 23988018. PMC3858465 23988018

[37] Feraco A. , Gorini S. , Camajani E. , Filardi T. , Karav S. , Cava E. , Strollo R. , Padua E. , Caprio M. , Armani A. , and Lombardo M. , Gender Differences in Dietary Patterns and Physical Activity: An Insight With Principal Component Analysis (PCA), Journal of Translational Medicine. (2024) 22, no. 1, 1112, 10.1186/s12967-024-05965-3, 39696430. PMC11653845 39696430

[38] Volaco A. , Cavalcanti A. M. , Filho R. P. , and Precoma D. B. , Socioeconomic Status: The Missing Link Between Obesity and Diabetes Mellitus?, Current Diabetes Reviews. (2018) 14, no. 4, 321–326, 10.2174/1573399813666170621123227, 28637406. 28637406

[39] Everson S. A. , Maty S. C. , Lynch J. W. , and Kaplan G. A. , Epidemiologic Evidence for the Relation Between Socioeconomic Status and Depression, Obesity, and Diabetes, Journal of Psychosomatic Research. (2002) 53, no. 4, 891–895, 10.1016/s0022-3999(02)00303-3, 12377299. 12377299

[40] Brown A. F. , Ettner S. L. , Piette J. , Weinberger M. , Gregg E. , Shapiro M. F. , Karter A. J. , Safford M. , Waitzfelder B. , Prata P. A. , and Beckles G. L. , Socioeconomic Position and Health Among Persons With Diabetes Mellitus: A Conceptual Framework and Review of the Literature, Epidemiologic Reviews. (2004) 26, no. 1, 63–77, 10.1093/epirev/mxh002, 15234948. 15234948

[41] van Dam R. M. , Willett W. C. , Rimm E. B. , Stampfer M. J. , and Hu F. B. , Dietary Fat and Meat Intake in Relation to Risk of Type 2 Diabetes in Men, Diabetes Care. (2002) 25, no. 3, 417–424, 10.2337/diacare.25.3.417, 11874924. 11874924

[42] Karnchanasorn R. , Huang J. , Ou H. Y. , Feng W. , Chuang L. M. , Chiu K. C. , and Samoa R. , Comparison of the Current Diagnostic Criterion of HbA1c With Fasting and 2-Hour Plasma Glucose Concentration, Journal Diabetes Research. (2016) 2016, 6195494, 10.1155/2016/6195494, 27597979. PMC4997021 27597979

[43] Kim S.-y. and Kang M. , Male Korean Workers Eating Out at Dinner, British Food Journal. (2018) 120, no. 8, 1832–1843, 10.1108/bfj-12-2017-0680.

