# Supplementary material for: Association of Family Meals and Prediabetes Among South Korean Adults Without Diabetes
Source: J Diabetes Res. 2026 Oct 14;2026:7457214. doi: 10.1155/jdr/7457214 (PMC13662959; doi:10.1155/jdr/7457214)
Supplement: Supplementary file 1 [file JDR-2026-7457214-s001.docx]

**Supplementary Figure 1. Flowchart of the study participants displaying the inclusion and exclusion**

**Supplementary Figure 2. Conceptual framework illustrating potential pathways and covariates in the association between family meal practices and prediabetes**

*: Body mass index was included as a prespecified covariate in the statistical models; however, its temporal relationship with family meal practices could not be established because of the cross-sectional study design.

| **Supplementary Table 1. Results of factors associated between family meals and prediabetes** | | | | | | | | | |
| --- | --- | --- | --- | --- | --- | --- | --- | --- | --- |
| **Variables** | **Prediabetes** | | | | | | | | |
|  | **Male** | | | |  | **Female** | | | |
|  | **OR** | **95% CI** | | |  | **OR** | **95% CI** | | |
| **Model 1** |  |  |  |  |  |  |  |  |  |
| **Family meals** |  |  |  |  |  |  |  |  |  |
| No | 1.00 |  |  |  |  | 1.00 |  |  |  |
| Yes | 1.24 | (1.10 | - | 1.40) |  | 0.97 | (0.87 | - | 1.08) |
| **Model 2** |  |  |  |  |  |  |  |  |  |
| **Family meals** |  |  |  |  |  |  |  |  |  |
| No | 1.00 |  |  |  |  | 1.00 |  |  |  |
| Yes | 0.83 | (0.72 | - | 0.96) |  | 0.98 | (0.85 | - | 1.11) |
| **Model 3** |  |  |  |  |  |  |  |  |  |
| **Family meals** |  |  |  |  |  |  |  |  |  |
| No | 1.00 |  |  |  |  | 1.00 |  |  |  |
| Yes | 0.85 | (0.74 | - | 0.98) |  | 0.97 | (0.84 | - | 1.11) |
| **Model 4** |  |  |  |  |  |  |  |  |  |
| **Family meals** |  |  |  |  |  |  |  |  |  |
| No | 1.00 |  |  |  |  | 1.00 |  |  |  |
| Yes | 0.84 | (0.73 | - | 0.97) |  | 0.97 | (0.84 | - | 1.11) |

Model 1 was unadjusted.

Model 2 was adjusted for demographic and socioeconomic covariates.

Model 3 was additionally adjusted for health-related behavioral covariates.

Model 4 was adjusted for all prespecified covariates.

| **Supplementary Table 2. Comparison of nutrient intake by family meal participation status** | | | | | | | | | | | | | |
| --- | --- | --- | --- | --- | --- | --- | --- | --- | --- | --- | --- | --- | --- |
| **Variables** | **Male** | | | | | |  | **Female** | | | | | |
|  | **Family meals** | | | | | ***P-value**** |  | **Family meals** | | | | | ***P-value**** |
|  | **Yes** | |  | **No** | |  |  | **Yes** | |  | **No** | |  |
|  | **N (%) / Median (IQR)** | | | | |  |  | **N (%) / Median (IQR)** | | | | |  |
| **Number of participants** | 4,898 (70.9) | |  | 2,006 (29.1) | |  |  | 7,269 (72.3) | |  | 2,778 (27.7) | |  |
| **Comparison to usual intake** |  |  |  |  |  | <.0001 |  |  |  |  |  |  | <.0001 |
| Increased | 718 (14.7) | |  | 343 (17.1) | |  |  | 1,176 (16.2) | |  | 543 (19.6) | |  |
| Similar | 3,746 (76.5) | |  | 1,421 (70.8) | |  |  | 5,214 (71.7) | |  | 1,878 (67.6) | |  |
| Decreased | 434 (8.9) | |  | 242 (12.1) | |  |  | 879 (12.1) | |  | 357 (12.9) | |  |
| **Energy, kcal** | 1,997.8 (1,580.7 - 2,536.0) | |  | 2,040.6 (1,541.0 - 2,657.5) | | 0.3610 |  | 1,492.8 (1,157.8 - 1,906.0) | |  | 1442.5 (1,094.5 - 1,880.8) | | <.0001 |
| **Water, g** | 1,035.8 (703.6 - 1,478.5) | |  | 1,028.1 (639.9 - 1,492.8) | | 0.0827 |  | 886.1 (604.5 - 1242.4) | |  | 822.1 (526.4 - 1,189.0) | | <.0001 |
| **Carbohydrates, g** | 288.4 (227.5 - 360.1) | |  | 277.9 (216.3 - 356.1) | | 0.0003 |  | 222.0 (171.7 - 282.7) | |  | 214.7 (162.1 - 278.2) | | 0.0002 |
| **Protein, g** | 74.3 (55.3 - 98.6) | |  | 75.0 (52.2 - 104.8) | | 0.5471 |  | 54.9 (40.5 - 73.6) | |  | 52.2 (36.2 - 70.8) | | <.0001 |
| **Total fat, g** | 45.7 (29.9 - 68.4) | |  | 48.7 (30.6 - 77.8) | | <.0001 |  | 35.9 (22.6 - 53.9) | |  | 33.7 (19.8 - 52.8) | | <.0001 |
| **Dietary fiber, g** | 26.9 (19.3 - 36.4) | |  | 23.4 (16.6 - 33.2) | | <.0001 |  | 21.2 (14.9 - 29.5) | |  | 20.1 (13.6 - 29.2) | | <.0001 |
| **Total sugars, g** | 53.0 (32.1 - 80.9) | |  | 51.8 (29.4 - 83.1) | | 0.4220 |  | 47.8 (29.5 - 72.6) | |  | 47.7 (27.7 - 75.2) | | 0.6751 |
| **Sodium, mg** | 3,485.8 (2,487.6 - 4,781.9) | |  | 3,438.3 (2,398.4 - 4,810.8) | | 0.3499 |  | 2,459.0 (1,679.5 - 3,440.7) | |  | 2,235.5 (1,454.7 - 3,240.6) | | <.0001 |
| **Potassium, mg** | 2,878.6 (2,150.7 - 3,766.4) | |  | 2,648.4 (1,927.5 - 3,569.0) | | <.0001 |  | 2,309.3 (1,693.0 - 3,119.0) | |  | 2,186.4 (1,520.7 - 3,042.6) | | <.0001 |
| **Magnesium, mg** | 326.4 (247.2 - 427.0) | |  | 295.9 (221.6 - 400.1) | | <.0001 |  | 253.2 (189.2 - 336.9) | |  | 239.0 (171.3 - 324.5) | | <.0001 |
| **Vitamin D, μg** | 1.8 (0.7 - 4.1) | |  | 1.7 (0.6 - 3.9) | | 0.0619 |  | 1.4 (0.5 - 3.1) | |  | 1.2 (0.4 - 3.0) | | <.0001 |

*: P-values were obtained using the chi-square test for categorical variables and the Wilcoxon rank-sum test for continuous variables, as the latter were confirmed to be non-normally distributed.

IQR: Interquartile range

| **Supplementary Table 3. Sensitivity analysis stratified by inclusion of the COVID-19 period (2020 and 2021)** | | | | | | | | | |
| --- | --- | --- | --- | --- | --- | --- | --- | --- | --- |
| **Variables** | **Prediabetes** | | | | | | | | |
|  | **Male** | | | |  | **Female** | | | |
|  | **OR** | **95% CI** | | |  | **OR** | **95% CI** | | |
| **Sample in COVID-19 period (2020, 2021)** | | |  |  |  |  |  |  |  |
| **Family meals** |  |  |  |  |  |  |  |  |  |
| No | 1.00 |  |  |  |  | 1.00 |  |  |  |
| Yes | 0.92 | (0.73 | - | 1.17) |  | 1.07 | (0.85 | - | 1.36) |
| **Sample not in COVID-19 period (2019, 2022, 2023)** |  |  |  |  |  |  |  |  |  |
| **Family meals** |  |  |  |  |  |  |  |  |  |
| No | 1.00 |  |  |  |  | 1.00 |  |  |  |
| Yes | 0.80 | (0.66 | - | 0.95) |  | 0.90 | (0.76 | - | 1.07) |

Adjusted for demographic, socioeconomic, health-related behavioral, clinical, and survey year covariates.
